# Immediate effects of acupuncture on biceps brachii muscle function in healthy and post-stroke subjects

**DOI:** 10.1186/1749-8546-7-7

**Published:** 2012-03-14

**Authors:** Ana Paula S Fragoso, Arthur S Ferreira

**Affiliations:** 1 Laboratory of Human Motion Analysis, Post-graduation Program of Rehabilitation Science, Centro Universitário Augusto Motta, Rio de Janeiro, Brazil; 2 Graduation Program of Physical Therapy, Universidade Salgado de Oliveira, Niterói, Brazil

## Abstract

**Background:**

The effects of acupuncture on muscle function in healthy subjects are contradictory and cannot be extrapolated to post-stroke patients. This study evaluated the immediate effects of manual acupuncture on myoelectric activity and isometric force in healthy and post-stroke patients.

**Methods:**

A randomized clinical trial, with parallel groups, single-blinded study design, was conducted with 32 healthy subjects and 15 post-stroke patients with chronic hemiparesis. Surface electromyography from biceps brachii during maximal isometric voluntary tests was performed before and after 20-min intermittent, and manual stimulation of acupoints *Quchi *(LI11) or *Tianquan *(PC2). Pattern differentiation was performed by an automated method based on logistic regression equations.

**Results:**

Healthy subjects showed a decrease in the root mean-squared (RMS) values after the stimulation of LI11 (pre: 1.392 ± 0.826 V; post: 0.612 ± 0.0.320 V; *P *= 0.002) and PC2 (pre: 1.494 ± 0.826 V; post: 0.623 ± 0.320 V; *P *= 0.001). Elbow flexion maximal isometric voluntary contraction (MIVC) was not significantly different after acupuncture stimulation of LI11 (pre: 22.2 ± 10.7 kg; post: 21.7 ± 9.5 kg; *P *= 0.288) or PC2 (pre: 18.8 ± 4.6 kg; post: 18.7 ± 6.0 kg; *P *= 0.468). Post-stroke patients did not exhibit any significant decrease in the RMS values after the stimulation of LI11 (pre: 0.627 ± 0.335 V; post: 0.530 ± 0.272 V; *P *= 0.187) and PC2 (pre: 0.601 ± 0.258 V; post: 0.591 ± 0.326 V; *P *= 0.398). Also, no significant decrease in the MIVC value was observed after the stimulation of LI11 (pre: 9.6 ± 3.9 kg; post: 9.6 ± 4.7 kg; *P *= 0.499) or PC2 (pre: 10.7 ± 5.6 kg; post: 10.2 ± 5.3 kg; *P *= 0.251). Different frequency of patterns was observed among healthy subjects and post-stroke patients groups (*χ*^2 ^= 9.759; *P *= 0.021).

**Conclusion:**

Manual acupuncture provides sufficient neuromuscular stimuli to promote immediate changes in motor unit gross recruitment without repercussion in maximal force output in healthy subjects. Post-stroke patients did not exhibit significant reduction on the myoelectric activity and maximal force output after manual acupuncture and needs further evaluation with a larger sample.

**Trial registration:**

Brazilian Clinical Trials Registry RBR-5g7xqh.

## Background

Deaths related to cardiovascular disease are expected to be 24 million in 2030 [1,2]. The importance of management of risk factors for stroke is getting reinforcement. The rehabilitation techniques are aimed to improve by minimizing both recovery time and neurologic impairments and maximizing functional independence of post-stroke patients [3,4].

The efficacy of Chinese medicine (CM) intervention in stroke-related functional impairments was evaluated. In the last two decades, most acupuncture controlled trials in post-stroke patients failed to obtain significant, long-term improvement of the functional aspect [5], suggesting that new methodological approaches were required, such as observation of neuromuscular activity [6-9]. Surface electromyography (sEMG) can evaluate muscle activity in health and morbid conditions in a noninvasive manner, allowing the assessment of the proportion and duration of muscular activity as well as the neural recruitment strategies. The root mean-squared (RMS) value of sEMG signals has been used as a time-domain parameter for monitoring changes in myoelectric activity since it reflects the relationship between muscle force and corresponding gross motor unit (MU) recruitment [10].

The immediate effects of acupuncture on the sEMG parameters were contradictory and not clearly understood in healthy subjects. Toma *et al. *[11] analyzed the sEMG signals of the flexor digitorum superficialis, flexor digitorum profundus, and semitendinous (n = 17; 20-38 years old). Interventions comprised of perpendicular needle insertion into these muscles during 15 min without specified acupoints. A significant increase in the sEMG responses was observed during maximal knee flexion but no significant difference was observed for the handgrip sEMG values. Tough [12] analyzed the sEMG activity of the common wrist extensor muscles (n = 35; 18-70 years old). All subjects arbitrarily received: (a) 20-min stimulation of *Deqi *true at acupoints *Hegu *(LI4) and *Shousanli *(LI10); (b) *Deqi *false or inappropriate in the acupoints *Quze *(PC3) and *Neiguan *(PC6); and (c) no stimulation (considered as a control). The results showed that sEMG was reliable (intra-class correlation coefficient = 0.9996), but no significant difference was observed among the protocols. Costa and Araújo [13] investigated the sEMG signals from the tibialis anterior muscle (n = 15 per group, 18-25 years old). The effects of manual stimulation at acupoints *Zusanli *(ST36) and *Yinlingquan *(SP9) were evaluated by the RMS values and maximal isometric voluntary contraction (MIVC) force estimated from sEMG and force signal, respectively. A significant reduction in the RMS values was observed in both ST36 and SP9 immediately after acupuncture, but the MIVC value was significantly reduced only after the stimulation at ST36. Therefore, as related to the sEMG signals derived from those muscles, the signal should increase in tonification and decrease in sedation. However, no convincing data could support this statement and no model was proposed to explain the observed results.

The immediate effects of acupuncture on the sEMG amplitude and MIVC values in post-stroke patients have not been investigated. Also, the results from healthy subjects cannot be extrapolated to post-stroke patients due to several reasons. According to CM theory, healthy subjects may present different frequency distributions of patterns and different responses to acupuncture, compared to post-stroke patients. From the biopsychosocial model, the structural lesion observed in post-stroke patients imposes a limited control of muscle activation leading to different strategies for MU recruitment. Thus, this study was justified by the severity of motor impairments and functional disability arising from stroke.

This study aims to evaluate the immediate effects of manual stimulation of acupoints on both electrical activity and strength of the biceps brachii muscle in two parallel groups (healthy subjects and post-stroke patients with chronic hypertonic hemiparesis); and to propose a model to explain the relationship between acupoint stimulation and variables of muscle function, *i.e*. the RMS and MIVC values.

## Methods

**Figure 1:**
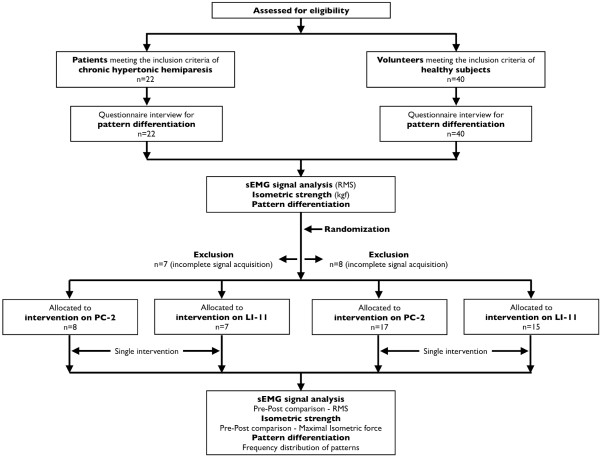
**Flowchart of the study**.

A detailed description of this randomized clinical trial, with two parallel groups (described as 'clinical study 1' and 'clinical study 2', respectively), single-blinded study design has been published [14]. The flowchart of this study design was depicted in Figure 1. This study protocol followed recommendations of both the Consolidated Standards of Reporting Trials [15], Standards for Reporting Interventions in Clinical Trials of Acupuncture [16], and Declaration of Helsinki. Also, the protocol was approved by the Institutional Committee of Ethics in research before execution (CAAE-0006.0.307.000-10). All subjects read and signed the written consent form after the explanation on the research aims and methods.

### Casuistic

Demographic characteristics were presented in Table 1.^Q1 ^In clinical study 1 (n = 40), healthy subjects were recruited from the academic institutional community. No pain, trauma or musculoskeletal injuries in the upper limbs, pregnancy, or any other contra-indication to acupuncture was found on them through the clinical screening. In clinical study 2 (n = 22), post-stroke patients were recruited from the Fluminense Rehabilitation Association. The patients with upper neurons lesion and a chronic (more than six months), partial motor impairment on the upper limb plus the above-cited characteristics of healthy subjects were included. The research included 32 healthy subjects and 15 post-stroke patients, and excluded all cases with incomplete the sEMG signal acquisition or sEMG low-quality signals.

**Table 1:** Clinical data from studied sample

	**Healthy subjects**			**Post-stroke patients**		
	
	**LI11**	**PC2**	***P***	**LI11**	**PC2**	***P***
				
**Sample size**	15	17		7	8	
Female	11 (73%)	15 (88%)		2 (29%)	5 (63%)	
Male	4 (27%)	2 (12%)		5 (71%)	3 (38%)	
**Age (y)**	24.1 ± 6.0	26.1 ± 6.6	0.381	50.9 ± 19.2	50.9 ± 0.3	0.126
**Weight (kg)**	66.5 ± 10.9	63.6 ± 11.9	0.480	74.6 ± 23.3	70.9 ± 25.1	0.666
**Height (m)**	1.64 ± 0.07	1.65 ± 0.06	0.940	1.70 ± 0.33	1.67 ± 0.32	0.388
**Heart rate (b/min)**	74.3 ± 11.1	75.8 ± 11.0	0.717	76.9 ± 25.5	81.8 ± 0.4	0.578
**Systolic pressure (mmHg)**	112.0 ± 16.1	114.7 ± 15.9	0.637	121.4 ± 37.1	132.5 ± 0.5	0.286
**Diastolic pressure (mmHg)**	65.3 ± 13.0	67.6 ± 10.3	0.586	80.0 ± 25.1	80.0 ± 0.0	1.000

### Setup for signal acquisition

Acupoints were located according to CM [17,18]. LI11 was located with the elbow flexed, the radial end of the elbow, at the midpoint of the line connecting *Chize *(LU5) to the lateral epicondyle of the humerus. PC2 was located on the medial side of the arm, 2 *cun *below the anterior end of the axillary fold, between long and short heads of biceps brachii muscle. LI11 and PC2 were cleaned with sterile cotton soaped with 70% alcohol and moistened with a 0.90% saline solution for the safety of patients and improvement of the sEMG signal conductivity. The sEMG signals were collected using an analog device EMG400C (EMG System, SP, Brazil) connected to a computer by a data acquisition card (NI-6009, 14 bits; National Instruments, Texas, USA). Disposable, auto-adhesive double-disk electrodes (Ag/AgCl; diameter = 10 mm; inter-electrode = 10 mm; Hal Industria, SP, Brazil) were placed according to the sEMG for the Non-Invasive Assessment of Muscles (SENIAM) recommendations [19] for the biceps brachii (short head). Active differential electrodes (gain: 20×) were used to collect the sEMG signals from surface electrodes to the analog device. Isometric force signals were acquired by a load cell (range: 0-200 kg; EMG System, SP, Brazil) connected to the same analog device synchronously to the sEMG signals at a sampling rate of 1.0 kHz per channel. Algorithms written in LabVIEW (National Instruments, Texas, USA) were developed to record and process the signals in the time domain.

### Procedures and intervention

All post-stroke patients answered the questions on their clinical status in a questionnaire form (Additional file 1) to differentiate their patterns developed based on [20]. Each volunteer was positioned in a chair with support for the upper limb to remain with the elbow at 90° (flexion) at the room temperature (21-23°C). Both acupuncture and signal acquisition were conducted by the same author (APSF) for the consistency of the procedure.

All volunteers executed three repetitions of isometric elbow flexion at MIVC during 5 s interleaved by 2 min interval to allow resting and metabolic recovery. In sequence, a sterile, disposable stainless-steel needle (0.20 × 13 mm, Lizhou, China) was inserted at an angle of 45° into the selected acupoint towards the direction of *Qi *circulation at a depth of approximately 1.5 cm (the needle length was chosen to minimize the depth of needle insertion among all volunteers). After insertion, the needle was rotated clockwise until the volunteer reported the first *Deqi*. In manual stimulation, clockwise needle rotation and it was performed immediately after the needle insertion, 5, 10, 15, and 20 min for 10 s, and accompanied by the report of *Deqi*. The stimulated traditional functions [21] decreased the RMS and MIVC values. After 20 min, the needle was removed and the signal acquisition was repeated. The surface electrodes were not removed during the acupuncture intervention to avoid the changes due to the electrode position relative to the innervation zone and improve inter-subject reliability.

### Reasons for acupoint selection

Two acupoints were selected for this research on the basis of the previous study [22] on the acupoint prescription for stroke-related disorders. Acupoint *Quchi *(LI11) was selected as the 'intervention acupoint' among the most cited acupoints. The location and traditional functions of LI11 are related to the motor impairments in post-stroke patterns [17]. Acupoint *Tianquan *(PC2) was selected as the active 'control acupoint' mainly because of its location.

### Signal processing and study outcomes

The primary outcomes of this study were the RMS and MIVC values estimated from sEMG and load cell signals, respectively. Signals were amplified by the analog device (gain: 2,000×) and digitally stored for off-line processing. Load cell signals were lowpass filtered (cut-off frequency: 5 Hz, Butterworth 2^nd ^order, direct and reverse order) and processed by an automatic, double-threshold method [23] that detected epochs of increased muscular force production for the estimation of MIVC value on each detected epoch. The maximum value was used as the representative value of MIVC at the respective test condition (pre or post intervention). The sEMG signals were bandpass filtered (cut-off frequencies: 5-450 Hz, Butterworth 2^nd ^order, direct and reverse order) and synchronously segmented with the load cell signal. All detected epochs were averaged to represent the RMS value at the respective test condition (pre or post intervention). In addition, the amplitude of a 5-s epoch of baseline signal was estimated with the RMS value for assessment of the lower boundary. The secondary outcome of this study was the frequency distribution of patterns in each group.

### Randomization and blinding

Four parallel groups were randomly generated using a web-based generator. Two sets of numbers (one per group) with 39 numbers per set in range 1-39 were sorted, annotated and inserted into sequentially numbered, opaque sealed envelopes before distribution to all volunteers. Each envelope contained the guidance of the acupuncture intervention: PC2 in the non-dominant arm of healthy subjects; LI11 in the non-dominant arm of healthy subjects; PC2 in the paretic upper limb; and LI11 in the paretic upper limb. Volunteers received their envelopes in order of admission to the study. The researcher opened the envelope to determine the guidance for acupuncture but the patient did not know the actual acupoint name and function. Additionally, the signals were stored without referring to the selected acupoint to allow a blinded signal processing of the sEMG data.

### Statistical analysis

Sample sizes were estimated from equations suggested to stroke-related outcomes [24]. A sample size of 36 subjects was calculated to observe the reduced RMS values on at least 80% of healthy subjects after the stimulation at LI11 (intervention acupoint) as compared to a 50% (random) probability in the same group after the stimulation at PC2 (control acupoint) considering α = 5% (Z_α _= 1.96; significance level) and β = 80% (Z_β _= 0.84; power of test). This *a priori *probability was chosen because: 1) needle stimulation on the control acupoint may have effects on RMS values; and 2) variables other than needle stimulation-such as sweating, cooperation-could reduce RMS values by chance in both groups. The RMS results from the healthy group were used to calculate the sample size for the post-stroke patients, yielding an estimated sample of 14 patients under the same α and β values.

Kolmogorov-Smirnov analysis showed that the RMS (average of the three repetitions) and MIVC values (average of the maximal value of each repetition) followed a Gaussian distribution. Intragroup (acupoint) analysis was conducted with unicaudal, paired student's *t*-test to test the null hypothesis that there was no difference in either the RMS or MIVC values between pre and post-intervention. Repeatability was tested with one-way analysis of variance for differences among the three repetitions of isometric contraction separately for acupoint group and test condition (pre and post-intervention). The items in the questionnaire for pattern differentiation were considered as dichotomous variables (present = 1; absence = 0) used as input variables for the regression equations for pattern differentiation and descriptive statistics of patterns was provided. Chi-square (*χ*^2^) test was used to test the null hypothesis of no difference in frequency distributions of patterns between healthy and post-stroke subjects. Statistical analysis was performed with SPSS^® ^software version 17 (SPSS Inc., Illinois, USA) and the significance level was considered at *P *< 0.05.

## Results

### Clinical study 1: Healthy subjects

The sEMG results and load cell signals were presented in Table 2. The RMS values significantly decreased after the stimulation at both LI11 (pre: 1.392 ± 0.826 V; post: 0.612 ± 0.0.320 V; *P *= 0.002) and PC2 (pre: 1.494 ± 0.826 V; post: 0.623 ± 0.320 V; *P *= 0.001). This result was not accompanied by a significant decrease in elbow flexion MIVC after the stimulation at LI11 (pre: 22.2 ± 10.7 kg; post: 21.7 ± 9.5 kg; *P *= 0.288) or PC2 (pre: 18.8 ± 4.6 kg; post: 18.7 ± 6.0 kg; *P *= 0.468). No significant difference was observed between LI11 and PC2 in the post-pre (Δ) values of MIVC and RMS (*P *= 0.340 and *P *= 0.391, respectively).

**Table 2:** Results from surface electromyography, maximal isometric voluntary contraction and pattern differentiation

	Healthy subjects		Post-stroke patients	
	**LI11**	**PC2**	**LI11**	**PC2**

**Maximal isometric voluntary force (kg)**				

Pre-intervention	22.2 ± 10.7	18.8 ± 4.6	9.6 ± 3.9	10.7 ± 5.6

Post-intervention	21.7 ± 9.5	18.7 ± 6.0	9.6 ± 4.7	10.2 ± 5.3

*P *(post × pre)	0.288	0.468	0.499	0.251

*P *(ΔMIVC LI11 × PC2)	0.340		0.303	

**Root mean square value (V)**				

Baseline noise	0.105 ± 0.010	0.115 ± 0.026	0.202 ± 0.085	0.179 ± 0.052

Pre-intervention	1.392 ± 0.826	1.494 ± 0.826	0.627 ± 0.335	0.601 ± 0.258

Post-intervention	0.612 ± 0.320	0.623 ± 0.320	0.530 ± 0.272	0.591 ± 0.326

*P *(post × pre)	0.002	0.001	0.187	0.398

*P *(ΔRMS LI11 × PC2)	0.391		0.220	

**Identified pattern**				

Fire heat	0 (0%)	3 (18%)	3 (43%)	4 (50%)

Phlegm dampness	7 (47%)	6 (35%)	2 (29%)	2 (25%)

*Qi *deficiency	7 (47%)	6 (35%)	1 (14%)	1 (13%)

*Yin *deficiency	1 (7%)	2 (12%)	1 (14%)	1 (13%)

*P *(healthy × patients)	χ^2 ^= 9.759; *P *= 0.021			

Repeatability analysis showed no significant difference on the RMS values from three repetitions of maximal voluntary effort before interventions on LI11 (*P *= 0.885) or PC2 (*P *= 0.892), as well as after the stimulation at those acupoints (*P *= 0.736; *P *= 0.906; respectively). Similarly, the MIVC force was also not significantly different among the three repetitions before the interventions on LI11 (*P *= 0.864) or PC2 (*P *= 0.977), as well as after the stimulation at those acupoints (*P *= 0.763; *P *= 0.986; respectively).

### Clinical study 2: Post-stroke patients

Post-stroke patients exhibited different results (Table 2). Pre-intervention, the MIVC values of all post-stroke patients were significantly reduced (50%; *P *< 0.001) compared to healthy subjects. The RMS values were not significantly decreased after the stimulation at both LI11 (pre: 0.627 ± 0.335 V; post: 0.530 ± 0.272 V; *P *= 0.187) and PC2 (pre: 0.601 ± 0.258 V; post: 0.591 ± 0.326 V; *P *= 0.398). This result was not accompanied by a significant decrease on MIVC after the stimulation at LI11 (pre: 9.6 ± 3.9 kg; post: 9.6 ± 4.7 kg; *P *= 0.499) or PC2 (pre: 10.7 ± 5.6 kg; post: 10.2 ± 5.3 kg; *P *= 0.251). No significant difference was observed between LI11 and PC2 in the ΔMIVC and ΔRMS values (*P *= 0.303 and *P *= 0.220, respectively).

Repeatability analysis showed no significant difference on the RMS values from three repetitions of maximal voluntary effort before the interventions on LI11 (*P *= 0.933) or PC2 (*P *= 0.750), as well as after the stimulation at those acupoints (*P *= 0.998; *P *= 0.731; respectively). Similarly, MIVC force was also not significantly different among the three repetitions before interventions on LI11 (*P *= 0.480) or PC2 (*P *= 0.970), as well as after the stimulation at those acupoints (*P *= 0.861; *P *= 0.881; respectively).

### Pattern differentiation

Significant different frequency distributions of patterns were observed among healthy and post-stroke patients (*χ*^2 ^= 9.759; *P *= 0.021; Table 2). Two patterns-"Phlegm dampness" and "*Qi *deficiency"-were the most commonly identified among healthy subjects (47% and 35%, respectively, on each acupoint group LI11 and PC2). Among post-stroke patients, pattern "Fire heat" was the most frequent one (LI11 group: 43%; PC2 group: 50%), followed by "Phlegm dampness", "*Qi *deficiency" and "*Yin *deficiency" patterns.

## Discussion

This study evaluated the immediate effects of the manual stimulation at acupoints on both myoelectric activity and strength of the biceps brachii in two parallel samples. The results showed that (1) manual acupuncture immediately decreased the RMS but not MIVC values in healthy subjects; (2) manual acupuncture did not immediately decreased RMS or MIVC values in post-stroke patients; (3) LI11 and PC2 elicited similar effects in both healthy and post-stroke groups; and (4) the frequency of identified patterns was different between two groups.

### Clinical study 1: Healthy subjects

The absence in significant difference among the three-repetition test suggested that the average values of RMS and MIVC were representative for further analysis in both parallel groups. The MIVC value of healthy subjects was similar to the previous study (189 N ≈ 19.3 kg) [25].

In the group of healthy subjects, the RMS values immediately decreased after the acupuncture, without a respective significant change in MIVC. These results were close to those reported by [13] where similar rationale for acupoint selection (specific indication *versus *location), methods for acupuncture stimulation (rotation every 5 min) and sEMG signal-processing details (RMS epoch duration = 5 s) were used. However, negative results were observed in studies [11,12] conducted with samples with similar characteristics but with different sEMG signal analysis and acupoint selection. Toma *et al. *[11] reported the use of an inter-electrode distance of 32 mm and evaluated the integration of sEMG signal during the entire range of motion divided by contraction time instead of the RMS values. Although the inter-electrode distance may be adequate to record the relative contributions of deep and superficial MU [26], it is larger than currently recommended (< 20 mm) [19]. Tough [12] used similar methods (sample size, intervention duration, *etc*.), acupoint selection (LI10 instead of LI11) and sEMG analysis (average of three trials, signal sampling frequency, statistical analysis, absolute amplitude values, *etc*.) except for the epoch duration (10 s each for the RMS estimation) and the lack of assessment of MIVC. Finally, the recommendations from the SENIAM project [19] for electrode positioning and signal processing were not reported [11-13] and may compromise external validity.

### Clinical study 2: Post-stroke patients

In the group of post-stroke patients, neither the RMS nor MIVC values were decreased after acupuncture. Clinical trials on acupuncture for post-stroke MU impairments often tested the efficacy of an intervention by functional outcomes [5-7], while few researches evaluated the physiologic effects of acupuncture intervention in post-stroke patients by electromyography. For instance, Zhao *et al. *[27] evaluated functional and electromyographic parameters (F-wave) and reported significant reduction in spasticity in chronic post-stroke patients submitted to 30 days of acupuncture due to reduced excitability of α-motoneurons. Yan and Hui-Chan [28] used functional scales, MIVC and electromyography (co-contraction ratio) in post-stroke patients with acute motor impairments and showed that three weeks of electroacupuncture significantly increased MIVC while co-contraction of medial gastrocnemius and tibialis anterior decreased. These results were attributed to the enhancement of presynaptic inhibition to the hyperactive stretch reflex and disinhibition of voluntary commands to the α-motoneurons of the paretic muscles. To the best of our knowledge, this may be the first study to present results on immediate effects of acupuncture stimulation on muscle function in post-stroke patients.

The lack of significant difference in this sample was attributed to a combination of at least four factors. First, RMS pre-intervention values were lower (42.4%) in post-stroke patients than healthy subjects probably as a consequence of a lower MIVC (50.0%)-confirming the negative features of upper limb paresis. This implies in a different "operational point" of the force-MU recruitment relationship and may have influenced the sEMG signal composite pattern. Second, as pre-intervention values were reduced and baseline noise was not different during the 20-min experiment, the possible range for reduction after intervention was compressed and a larger sample might be necessary to detect any possible significant difference, if existent. Third, the neurophysiology of MU recruitment is different for the motor impairments of post-stroke patients [29]. Fourth, neuromuscular activation and muscle unloading were reduced in chronic hemiparetic patients with retained neuromuscular connectivity, which lead to changes in fiber type composition (mainly type-II fibers) in the affected limb [30]. Therefore, the results of this study should be considered as preliminary until a large-sample study confirms the results in this study. Also, it is suggested to perform pre and post-intervention comparisons on smaller percentages of MIVC (*e.g*. 50%) to evaluate the effect of the above-cited issues.

### A model for sEMG and MIVC interpretation

Currently, the physiologic explanation for the observed neuromuscular behavior was limited to a "reflex loop" hypothesis and did not have clear explanations on physiologic mechanisms and enrolled structures [11-13]. Based on the current results and consideration on the sEMG signal characteristics and neurophysiology, a theoretical model was proposed for explanation of the results in healthy subjects that was compatible to the adaptive changes in post-stroke subjects and considered acupuncture technique.

Initially, insertion of the acupuncture needle 15 mm deep into the tissue penetrates the dermis and subcutaneous muscles and creates a small wound (needle radius = 0.2 mm) with probable fluid exudation [31]. Rotation of the needle following insertion promotes the mechanical coupling between the needle and connective tissue, and causes winding of tissue surrounding the needle [32,33]. This mechanical signal (passive deformation) is transmitted to connective tissue cells and is amplified due to increased tissue displacement [31-33]. The superficial area of tissue deformation may reach 25 mm^2 ^or more around a single needle hole [33] and stimulates several afferent nerve types, evidenced by the variety of subjective report of the *Deqi *sensation [34] most frequently described as "aching" or "soreness" [35]. CM theory states that *Deqi *must be achieved and sustained throughout the session to maximize therapeutic effects and thus needle is intermittently manipulated at short-time intervals (3 to 5 min). Finally, a significant higher pullout force is necessary for needle extraction from real acupoints [36] with additional tissue injury due to connective tissue adherence to the needle tip. Altogether, CM acupuncture intervention (= needle insertion + intermittent manipulation with *Deqi *+ needle extraction) evokes an uncomfortable sensation during the entire session and sometimes is prolonged after intervention.

Regarding the sEMG signal interpretation, it was reported that decreased MU discharge rate with pain was accompanied by changes in the population of MU used to maintain force [37]. Moreover, pain decreased MU synchronization with a consequent decrease in the sEMG amplitude estimators, mainly due to amplitude cancellation between positive and negative phase of the MU action potential [38]. Hence, the decreased RMS (healthy group: -56% to -58%; post-stroke patients: -2% to -16%) and sustained MIVC (healthy group: 0% to -2%; post-stroke patients: 0% to -5%) values observed after the stimulation at both LI11 and PC2 were fully consistent with the proposed model that the sustained painful sensation may decreased MU discharge but does not significantly changed MIVC. The lack of acupoint specific effects was also reported by other studies with sEMG and clinical outcomes [11,12,39] and was consistent with the results of the present study and the proposed model. Further studies are necessary to validate the proposed model, especially if the long-term effects were attributed to multiple short-term acupuncture stimulation on the sEMG variables.

### Methodology

As an important muscle commonly affected in upper neuron lesions, the biceps brachii is well suited for sEMG analysis because of its long, parallel fibers with a main innervation zone often located at the muscle belly. Although our parallel groups exhibit different characteristics on gender and age at baseline, a recent study found no significant effect of gender and age (range: < 10 to 70 years old) subjects regarding electromyographic parameters obtained from the biceps brachii [40] and thus we believe that this factor had no major influence on the obtained results. The RMS value fails to yield a general relationship with muscle strength [40]. Hence, changes in the RMS values of sEMG after an intervention may not rigorously reflect any altered level of neural drive to the muscle [41]. Additionally, a recent study [42] have demonstrated that the high frequency band (> 440 Hz) of sEMG signals allows an accurate estimation of the force-RMS relationship and needs further attention to assess the robustness of the proposed model to changes in the sEMG processing techniques.

### Pattern differentiation

In the present study, CM pattern differentiation was performed by an automated model designed for post-stroke patients from a large sample study [20]. The interesting result was that despite the small sample size of post-stroke patients, this study supported the CM theory that different frequency distributions of patterns may occurs between them and healthy subjects. However, a larger study sample and a proper study design may be necessary to provide more definitive conclusions. Nevertheless, the obtained results may be used to design studies on pattern differentiation, *e.g*. determining sample sizes based on prevalence of patterns. As a limitation, the binary logistic regression method was not validated and its diagnostic accuracy is unknown. Moreover, it was not yet applied to a sample of healthy subjects until now. Ongoing research on this topic included the determination of diagnostic accuracy of the method in post-stroke patients, the establishment of cutoff points for probabilities estimates from each regression equation, and comparison with other automated methods for pattern differentiation [43-45]. As it was argued that pattern differentiation may lead to best therapeutic results [46], the automated model with highest diagnostic performance should be used in future clinical trials to determine the therapeutic intervention in a reproducible manner.

## Conclusion

Manual acupuncture provides sufficient neuromuscular stimuli to promote immediate changes in MU gross recruitment without repercussion in maximal force output in healthy subjects. Post-stroke patients did not exhibit significant reduction on myoelectric activity and maximal force output after manual acupuncture and needs further evaluation with a larger sample.

## Abbreviations

CM: Chinese medicine; sEMG: Surface electromyography; RMS: Root mean-squared value; MU: Motor unit; MIVC: Maximal isometric voluntary contraction; ΔRMS: Difference between post and pre RMS values; ΔMIVC: Difference between post and pre MIVC values; SENIAM: Surface electromyography for the non-invasive assessment of muscles.

## Competing interests

The authors declare that they have no competing interests.

## Authors' contributions

APSF designed the study, conducted the questionnaire interview, selected the subjects, and wrote the manuscript. ASF designed the study, developed the computational methods for pattern differentiation, performed the statistical analysis, and wrote the manuscript. All authors read and approved the final version of the manuscript.

## Supplementary Material

Additional file 1 **Questionnaire for assessment of eligibility and pattern differentiation**. This illustration presents the complete form grouped by: subject's identification; clinical characterization; inclusion criteria; exclusion criteria; group and acupoint assignment; and manifestations regarding patterns distributed among the Four Examination. Click here for file
