# Supplementary material for: Immediate effects of acupuncture on biceps brachii muscle function in healthy and post-stroke subjects
Source: Chin Med. 2012 Mar 14;7:7. doi: 10.1186/1749-8546-7-7 (PMC3361469; doi:10.1186/1749-8546-7-7)
Supplement: Additional file 1 — Questionnaire for assessment of eligibility and pattern differentiation. This illustration presents the complete form grouped by: subject's identification; clinical characterization; inclusion criteria; exclusion criteria; group and acupoint assignment; and manifestations regarding patterns distributed among the Four Examination. [file 1749-8546-7-7-S1.PDF]

Stroke-related patterns in Chinese medicine

Screening form for pattern differentiation

1. Identification

1.1 ID number: ____	1.4 Weight (kg): ____	1.7 Gender: ☐ Male ☐ Female
1.2 Name (initials): ____	1.5 Height (m): ____	1.8 Dominant side: ☐ Right ☐ Left
1.3 Age (y): ____	1.6 Heart rate (bpm): ____	1.9 Blood pressure (mmHg): _____/_____

2. Clinical characterization

2.1 Etiology: ☐ Ischemic ☐ Hemorrhagic	2.6 Location: ________________________
2.2 Affected side: ☐ Right ☐ Left	2.7 Time after first stroke (months): ______
2.3 Orientation: ☐ Yes ☐ No	2.8 Tone of the affected limb:
2.4 Lucidity: ☐ Yes ☐ No	☐ Hypertonia ☐Hypotonia ☐ Eutonia
2.5 Communication: ☐ Yes ☐ No	2.9 Active elbow flexion:
	☐ Total-0° ☐ Total-90° ☐ 90°-0°

3. Exclusion criteria

3.1 Myopathies ☐ Yes ☐ No	3.5 Serious peripheral nerve injury ☐ Yes ☐ No
3.2 Poor Nutrition ☐ Yes ☐ No	3.5 Serious peripheral circulatory injury ☐ Yes ☐ No
3.3 Uncontrolled hypertension ☐ Yes ☐ No	3.7 Severe cardiopulmonary disease ☐ Yes ☐ No
3.4 Coagulation disorders ☐ Yes ☐ No	3.8 Acute inflammation in affected arm ☐ Yes ☐ No

4. Group and intervention

Group: ☐ Post-stroke ☐ Healthy	4.2 Intervention: ☐ PC2 ☐ LI11

5. Inspection

5.1 Overweight [1]: ☐ Yes ☐ No	5.2 Flushed cheek: ☐ Yes ☐ No	5.3 Eye congestion: ☐ Yes ☐ No

5.4 Complexion:	☐ Pale	☐ Yellow	☐ Reddened	☐ Darkish	☐ N/A	
5.5 Tongue color:	☐ Pale	☐ Pale red	☐ Reddened	☐ Bluish-purple	☐ N/A	
5.6 Tongue body:	☐ Mirror	☐ Spotted	☐ Ulcerated	☐ Teeth-marked	☐ Enlarged	☐ N/A
5.7 Tongue Fur:	☐ White	☐ Yellow	☐ Dry	☐ Thick	☐ N/A	

6. Auscultation-Olfaction

6.1 Phlegm rale: ☐ Yes ☐ No	6.2 Faint low voice: ☐ Yes ☐ No

7. Inquiry

7.1 Eyeball dryness ☐ Yes ☐ No	7.11 Insomnia ☐ Yes ☐ No
7.2 Constipation ☐ Yes ☐ No	7.12 Fatigue ☐ Yes ☐ No
7.3 Chest discomfort ☐ Yes ☐ No	7.13 Borborigmus ☐ Yes ☐ No
7.4 Heat in the palms and soles ☐ Yes ☐ No	7.14 Nausea ☐ Yes ☐ No
7.5 Afternoon tidal fever ☐ Yes ☐ No	7.15 Halitosis ☐ Yes ☐ No
7.6 Reversal cold of the extremities ☐ Yes ☐ No	7.16 Night sweating ☐ Yes ☐ No
7.7 Frequent urination ☐ Yes ☐ No	7.17 Profuse sweating ☐ Yes ☐ No
7.8 Aversion to heat ☐ Yes ☐ No	7.18 Headache ☐ Yes ☐ No
7.9 Vexing heat in the extremities ☐ Yes ☐ No	7.19 Bitter taste in the mouth ☐ Yes ☐ No
7.10 Reddish yellow urine ☐ Yes ☐ No	7.20 Thirsty ☐ Yes ☐ No

8. Palpation

8.1 Depth: ☐ Floating ☐ Deep ☐ N/A	8.4 Width: ☐ Thin ☐ Flooding ☐ N/A
8.2 Strength: ☐ Strong ☐ Vacuous ☐ N/A	8.5 Speed [2]: ☐ Rapid ☐ Slow ☐ N/A
8.3 Quality: ☐ Slippery ☐ Rough ☐ N/A	

[1]BMI>23 kg/m^2; [2]Fast>5 pulses/ breathing; Slow<4 pulses/breathing; N/A: none of the answers.
